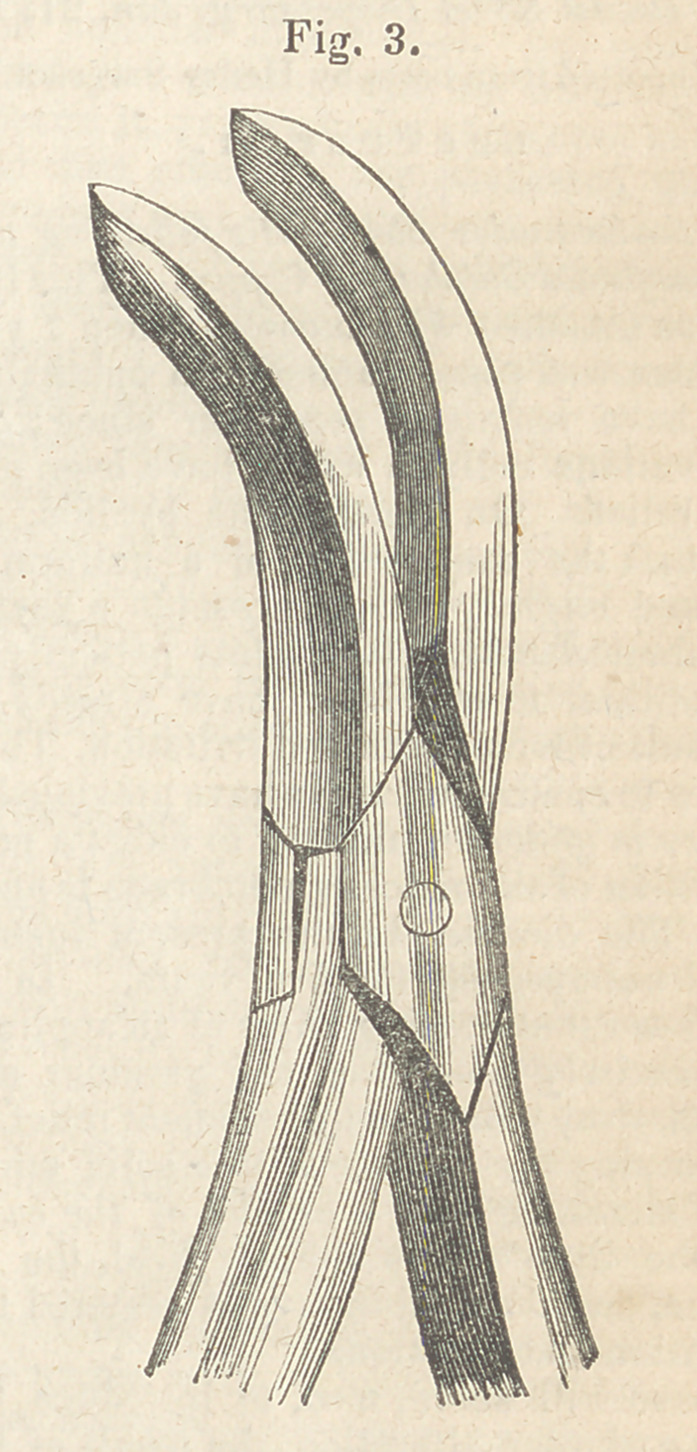# Jefferson Medical College

**Published:** 1843-02-18

**Authors:** H. T. Child


### CLINICAL LECTURES AND REPORTS.

### JEFFERSON MEDICAL COLLEGE.

#### CLINIC OF PROFESSOR MÜTTER.

*Dispensary of Jefferson Medical College, Jan.* 11, 1843.

#### LECTURE II.

(Reported by H. T. Child.)

(Concluded.)

Case II.—*Epulis, developed in the substance of the gum, and originating in neglected Parulis,*—The patient, a female, aged 35, had suffered from the presence of the disease for the last twelve months. The tumour belonged to the class of "spongy epulis," and equalled in size a small walnut. It bled from the slightest cause, and was occasionally painful; the patient's general health was excellent, and the glands about the neck were not at all affected. Professor M. removed the tumour by passing a tenaculum through its substance in order to have it completely under control, and then with a probe-pointed bistoury separated it from its attachments. The teeth and bone in the vicinity being sound, it was deemed sufficient to limit the operation to the excision of the tumour. The hæmorrhage was profuse, and after the usual agents, pressure and styptics, had failed to arrest it, the actual cautery was applied, which at once accomplished the object in view.

The term epulis, derived from two Greek words, (επι, upon, and ουλα, the gums,) has been applied to tumours of different kinds which are developed in the gums, membranes of the teeth, periosteum of the alveoli, the surface or internal structure of the bones, or the membranous lining of their cavities. The proximate causes of such growths are for the most part obscure, but many cases may be traced to carious teeth, neglected parulis, by which the bone was rendered carious or necrosed, blows upon the part, and fractures of the bones, as in the cases of Marjolin and Berrard, the chewing of acrid substances, as bad tobacco, and finally to some constitutional taint.

These tumours presenting different appearances and producing altogether dissimilar results, have been divided into distinct groups.

1st. The variety most commonly met with, is that characterized by the developement upon the gum, between the roots of the teeth, or in the alveolar socket, of a red, soft spongy mass, but slightly sensitive, and bleeding from the slightest touch; the constitution is but slightly, if at all involved, and the tumour is generally curable, but often returns when removed, and thus causes much anxiety to the patient. If neglected or treated with caustics it may assume a cancerous, or rather, a malignant action, and ultimately prove fatal. When the tumour originates in the alveolar cavities, the first indications of the disease are the loosening of the teeth which are perfectly white and sound, swelling of the gums, and a slight discharge of pus. This form of epulis is sometimes described as "Fungous of the Gums," and may be referred in many cases to carious teeth or injuries of the bone.

2d. A tumour very similar in appearance, but characterized in its commencement by pain, a foul and acrid discharge, disposition to ulcerate and bleed, is also found in the same spot. This is genuine cancer, and is one of the most intractable and obstinate of all the diseases of the gums.

3d. Another variety of epulis is characterized by a tumour, the tissue of which is much firmer than that composing the first and second kinds. The swelling is also beneath the mucous membrane, is usually smooth, very red, elastic, more or less compressible, and often pulsatile; affording no hæmorrhage, unless wounded, and possessing but little sensibility. When cut into, these tumours bleed freely, and when excised, present all the peculiarities of erectile tissue. They may be the result of injuries—but usually appear without any appreciable cause, and occasionally degenerate into malignancy.

4th. A fourth form of epulis is known by its roughness, hardness, pale and livid colour denoting slight vascularity, and often severe lancinating pain. The latter symptom is not, however, invariably present, and generally indicates, when met with, a malignant disposition in the tumour. It is this form from which the most danger is to be apprehended. Like all other tumours composed of this tissue they may remain indolent for many years, but depending as they usually, but not invariably do, upon a constitutional taint, such a result is not to be anticipated.

When either of the tumours just described, after having remained stationary for a longer or shorter period, takes on a new action, and grows rapidly, a new series of phenomena is at once developed. The teeth are loosened, the bones, if not before diseased, become carious, fungous masses of large size shoot up; the tumour, if originally hard, softens, a fœtid, purulent and bloody discharge is established, neighbouring parts, especially the lymphatic glands, are speedily involved in the disease, the whole constitution gradually gives way, and finally death, occasioned either by hectic, hæmorrhage, or suffocation, closes the scene.

The lower jaw is the most common seat of epulis, although the upper is by no means exempt from it; usually the tumour occupies the anterior portion of the bone, but occasionally it is seen on the sides or very far back. Its form and size varies, some are circumscribed with pediculated attachments, others have extended bases, and are lost in the adjacent parts, while in size there is the greatest diversity.

The prognosis will depend entirely upon the nature of the tumour, its size and disposition to increase. When of the first class and dependent upon carious teeth or bone, of moderate size, and with little or no disposition to spread, the case is one of easy management. Erectile epulis, although more serious in its character than the first kind, is yet a disease susceptible of cure, and rarely degenerates into cancer. But when the tumour is hard and fibrous accompanied by pain, and increases gradually, although certainly, and the constitution becomes involved, the case is one requiring all our attention, and often proves indomitable. The same may be said of the second variety of Epulis. Mr. Liston is under the impression that a genuine malignant tumour of the gum is *rare*, and in this I agree with him.

I have removed a great number of tumours of the jaws, in fear of their becoming malignant, and to relieve the patient's mind as much as any thing else, and have rarely failed in making a complete cure.

The only diseases with which epulis can be confounded are parulis, exostosis, spina ventosa, osteo sarcoma, and periosteal tumour, the result of inflammation. The history of the case, with an examination into the symptoms, will be sufficient to enable us to arrive at a correct diagnosis.

The treatment of this affection is of course modified by its nature and extent. Where it is merely a fungous mass arising from a carious tooth, or bone, or from necrosis, the indication is to remove the exciting cause, and then either pare off the tumour with the knife or scissors, or keep it down with cauteries and pressure. I prefer the knife, and if the granulations sprout they may be suppressed by caustic or astringents. The hæmorrhage which always takes place after the use of the knife may be arrested by compression or styptics, or the cautery. Erectile epulis, if pediculated, may be removed either by the knife or ligature, but where the base is extended its removal will require a much more extensive operation, for no part of the disease is to be left behind. The most profuse hæmorrhage is sure to be developed by cutting out such a tumour, and the actual cautery is usually required for its arrestation. If the tumour is small, compression with lint steeped in creosote or muriated tincture of iron will sometimes be sufficient to arrest the hæmorrhage.

But whenever the tumour is hard, knotty and painful, or spongy and painful, in other words, presents traces of scirrhosity or cancerous action, a very different operation has to be performed, if we desire to eradicate the disease.

Nothing short of the complete removal of the tumour, teeth, and bone connected with it, and the application of the actual cautery to the surface from which we take them, will accomplish our object. For removing such tumours some propose the chisel and mallet, but the operation best adapted to the case, and one which will occasion the least suffering is the following:

Having placed the head of the patient in a good light, and against the chest of an assistant who stands behind the operating chair, the surgeon makes a perpendicular incision on each side of the tumour with a pair of strong scissors, or rather cutting forceps, (*fig.* 1.) and without stopping to arrest the

Fig. 1.

hæmorrhage, at once detach the mass by dividing the alveolar process *above* or *below* the tumour, as the upper or lower jaw happens to be involved, with cutting forceps, (*fig.* 2.) one blade of which is ap-

Fig. 2.

plied to the inner portion of the jaw, and the other to the outer. The raw surface is next carefully examined, and every vestige of the disease removed with the knife or scissors. If the bone appears affected it must be cut away, and often a very good preventive to a return of the disease is the use of the actual cautery. The bleeding may be arrested either by the cautery, or by placing a pledget of lint dipped in creosote, in the chasm over which a slice of cork may be laid and then closing the jaws, make the sound one act as a compress. To secure the dressing the bandage for fracture of the lower jaw may be applied. The parts should not be examined in the first twenty-four hours, but after this period a daily dressing is required, the nature of which depends on circumstances. If there be no disposition to a return of the disease, it will be sufficient to wash the parts with some detergent solution, until cicatrization is completed. But if fungous granulations make their appearance, the vegetable caustic, or actual cautery should be applied, until this disposition in the wound is destroyed. When the healing process is finished the deformity occasioned by the loss of bone and teeth can be readily removed by the introduction of false gums and teeth.

Instead of making the perpendicular cuts with the short forceps, some prefer a thin saw, but the method just described is less painful, and accomplishes the object in a much shorter period. Where the tumour is very large the saw may be required, and should always be at hand in case the edges of the forceps should turn in making the first cut.

Where the tumour is very large or seated on the side of the bone, or far back, the cheek should be divided in order to enable us to reach it without difficulty. In such cases I have found great advantage in using a cutting forceps (*fig.* 3.) so curved

Fig. 3.

as to pass readily to the back part of the mouth, and then allow of the blades being passed above the base of the tumour, when the upper jaw is affected, and below it when the inferior maxillary is the seat of the disease.

After the removal of the diseased mass the wound of the cheek must be closed, and union by the first intention attempted, and where the dressings are carefully attended to, the deformity resulting from the incision is scarcely perceptible, unless the *portio dura* is extensively injured, when paralysis generally incurable ensues, often giving rise to a very unpleasant expression of countenance.

Professor M. then exhibited a specimen of fibrous tumour of large size, which he had recently removed from a patient of Dr. Fromville's, of Montgomery county, in this state. In the operation cutting forceps of different construction were employed, and it was necessary to remove a large portion of the upper maxillary bone. The hæmorrhage was arrested by the cautery. He mentioned that he had performed the operation of removing the upper maxillary either in part or completely, five times, and had always used the forceps in preference to the mallet and chisel, or saw. He also stated that this bone had been removed, as well as large portions of the lower jaw, first by Gensoul, of Lyons; Graefe of Berlin; Dupuytren, of Paris; Sir Philip Crampton and Cusack, of Dublin; Syme, of Edinburgh; and Liston, of London, and recently by many others at home and abroad. These operations are, however, of recent introduction into surgery, and richly deserve being ranked among the most important and useful of our science.

## Figures and Tables

**Fig. 1. f1:**
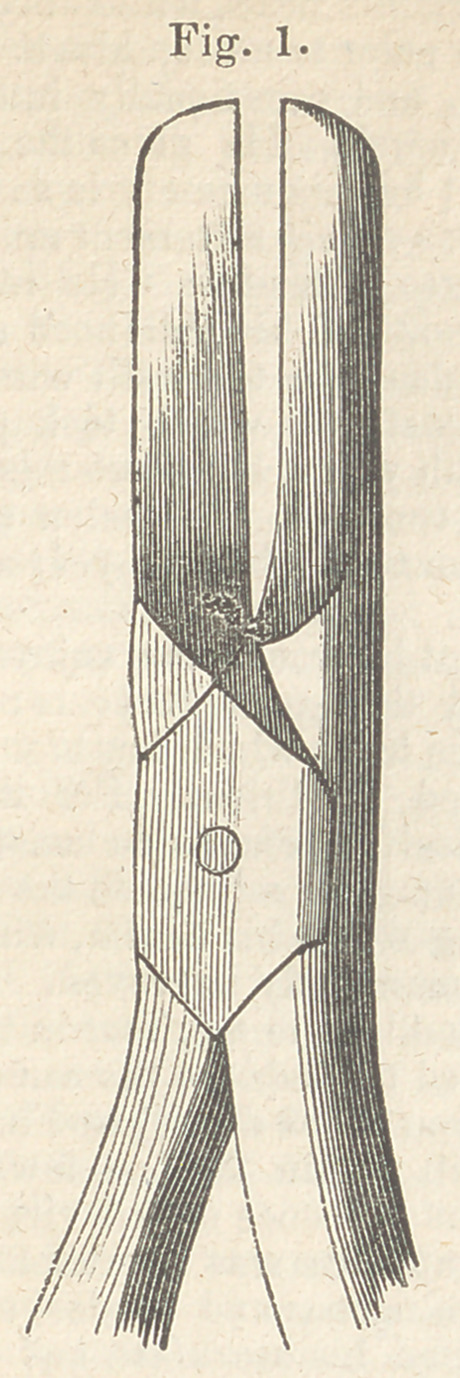


**Fig. 2. f2:**
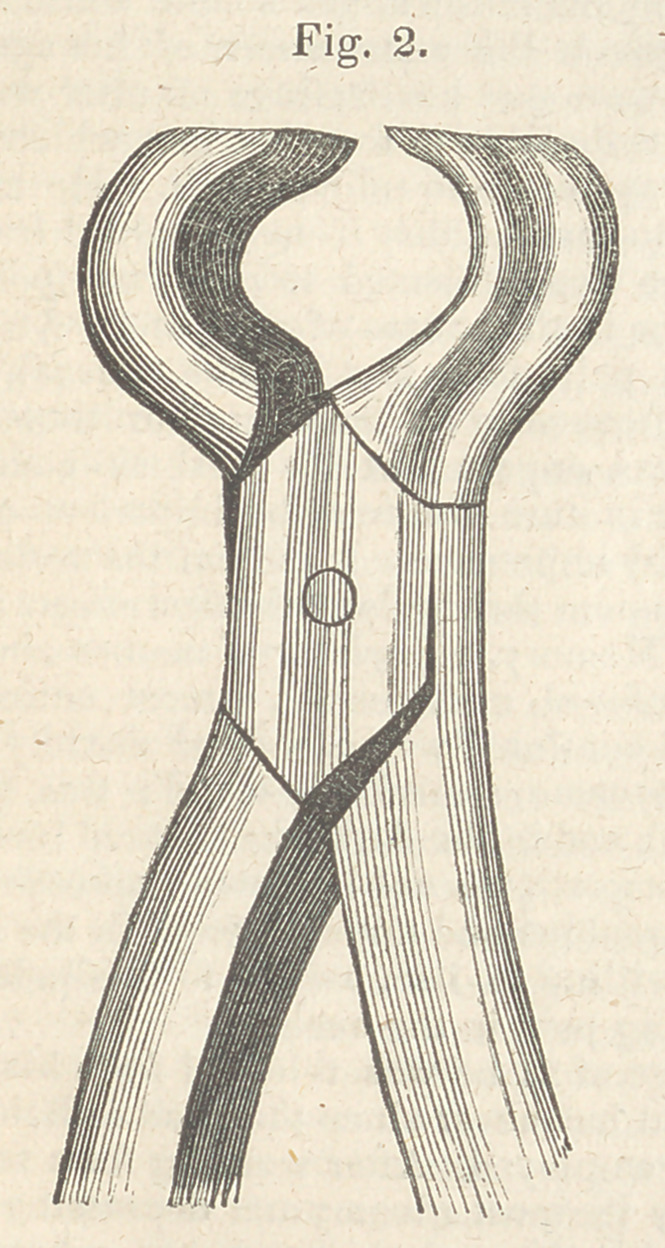


**Fig. 3. f3:**